# 3D printed orthopedic prostheses for domestic and wild birds—case reports

**DOI:** 10.1038/s41598-024-58762-9

**Published:** 2024-04-05

**Authors:** Lucas Rannier R. A. Carvalho

**Affiliations:** 1 Department of Physiology and Pharmacology, Karolinska Institutet, Biomedicum, 5B, Solnavägen 9, 171 65 Stockholm, Sweden; 2 Department of Veterinary Sciences, University Center of João Pessoa – UNIPÊ, João Pessoa, Brazil

**Keywords:** 3D technology, Conservation, Wildlife, 3D prostheses, FDM technology, Animal biotechnology, Biological techniques, Animal physiology

## Abstract

Regardless of the species, birds are exposed to injuries that lead to amputation of part of the body structure and often euthanasia. Based on the need for new technologies that improve the quality of life of birds with locomotor problems, the present case reports aimed to describe the development of custom-made three-dimensional (3D) prostheses for domestic and wild birds that suffered amputation or malformation of the hind limb. Using the measurements of the bird, a digital model was created for 3D printing using fused deposition modeling technology (FDM) by the Brazilian company 3D Medicine. In this study we report the use of 3D prosthesis for the rehabilitation of three birds with locomotor disorders in Brazil, the animals adapted to the custom-made prosthesis with an improvement in quality of life, better distribution of body weight, locomotion, and landing. This study describes the development of 3D prostheses for birds in Brazil, the first report of this technology for these species, and the pioneering development of socket prostheses for small birds. 3D prostheses offer a high-efficiency solution to improve the quality of life of animals with amputations and malformations of the hind limbs. In addition, 3D technology provides valuable tools for veterinary medicine, developing custom-made models for the most different anatomical demands of animal patients.

## Introduction

According to the European College of Zoological Medicine^1^, avian medicine is a distinct specialized field within veterinary medicine, focusing on the treatment of all avian species. Expanding medical care beyond poultry production. In Brazil, this sector is further subdivided into three main branches:

1. *Poultry production*: Brazil is the second largest producer and largest exporter of chicken meat in the world, with more than 14 million tons produced in 2022. In addition, the country has an important production of eggs, turkey, duck, derivatives, and the growing export of poultry genetic material^2^.
2. *Pet Market for Companion Birds*: Brazil has the second largest population of dogs, cats, and birds in the world and is the third largest in terms of total population of pets. It is estimated that in 2022, there were more than 41 million songbirds and ornamental birds in Brazil, almost 25% of all pets in the country^3^.
3. *Wild Birds*: One of the richest biodiversity in the world is the Brazilian. With almost 2,000 species, the Brazilian bird fauna represents approximately 20% of all identified species in the world^4^. In addition to the number of species, birds have an incredible variety of phenotypic characteristics, plumage, song patterns and geographic distributions. This rich fauna makes the Brazilian territory a major target of animal trafficking, with birds suffering most from criminal trade^5^.

In avian medicine, the incidence of amputations of wings, beaks and hind limbs is increasing. The main causes that lead to amputations are infectious diseases, physical trauma, and neoplasms^6^. In birds, physical injuries can be observed in flying accidents (colliding with walls), being run over on highways and mainly by inappropriate transport and accommodation when illegally trafficked^7^. Additionally, congenital malformations that lead to functional and structural alterations of the limbs and beak are also observed^8^.

Birds with congenital or acquired changes in the musculoskeletal system most often have severe difficulty in locomotion, flight and feeding. In the clinical routine, for severe cases euthanasia is recommended, in rare cases the animal has an amputated wing or limb and lives with limitations. For poultry production, euthanasia is even more frequently performed^9^.

Practical, quick, and low-cost solutions are not readily for avian medicine. However, with the continuous growth of different medical and veterinary healthcare sectors, the development of prostheses for birds has gained more attention, even with the significant difference between sizes and anatomical and behavioral characteristics. Literature on the use of prostheses in avian medicine is scarce; however, in recent years, prostheses for beak^6,10^, wings^9^, and hind limbs^11–13^ have been developed.

In this context, three-dimensional (3D) printing technology offers an interesting option for the development of prosthetics and orthoses for birds. By definition, *prostheses* are models that fully or partially replace a limb or organ, while *orthoses* are models that assist the limb, organ, or tissue, avoiding or controlling deformities, in addition to compensating for possible functional exceptions^14^.

Using the patient's measurements, 3D scanner or computerized tomography (CT) scan, it is possible to create a digital model of the prosthesis with high precision. The file is custom-made based on the anatomical characteristics of the amputated/malformed region and can be materialized by different methods and materials, such as nylon, metal, resins, and different types of organic or nonorganic plastics^15,16^.

The aim of this work is to describe the development of 3D prostheses for domestic and wild birds in Brazil. Combining solutions for cases of amputations and malformations using novel methods of 3D modeling and printing technology.

## Methods

Three-dimensional printing refers to the process of creating physical objects from digital models. The virtual file can be built using different tools such as: (1) computer-aided design (CAD) software using a 3D modeling program; (2) scanning a preexisting model or the patient's own structure using 3D scanners and (3) using medical scanning techniques such as magnetic resonance imaging (MRI) or computerized tomography (CT scan) and subsequent conversion to a 3-dimensional file^17,18^.

These tools used together or separately can optimize the process of creating the digital model that will be materialized by 3D printing. In addition, it is possible to use the virtual models for studies and simulations using augmented reality (AR) even before^19,20^. The 3D digital models in this work were produced using a combination of 3D scanning technologies (Revopoint Pop Scan 2, 10 fps, accuracy up to 0.05 mm, Revopoint 3D Technologies Inc, Hong Kong, China) and the 3D modeling software Blender® version 3.6^21^, generating a final file in STL format (acronym meaning “stereolithography”, one of the 3D printing methods).

The file in STL format can then be imported into the configuration software for 3D printing, known as the heart of the workflow. These programs are called “slicers” due to the function of converting the virtual model data into a file of transverse layers, which guide the movement of the material deposited through the printer (FDM printers). The final file exported by the slicer and interpreted by the machine is in not editable G-CODE format. This is a computer numerical control (CNC) format used mainly in computer-aided manufacturing to control automated machine tools^22^.

To generate the final G-CODE file, it is necessary to input machine data such as model, print nozzle and type of polymer used in the slicer software. In addition, the parameters for the operation of the printer, such as nozzle temperature, printing speed, retraction, towers to support the physical model, infill and wall thickness, which will determine the final quality of the physical model.

To prepare the orthopedic prosthesis for this study, Ultimaker Cura® 5.3.0 slicer software (Ultimaker, Netherlands) was used for printing by the fused deposition modeling (FDM) method an additive manufacturing (AM) process, where the plastic filament is melted and deposited under pressure according to the coordinates^15^.

**Figure 1:**
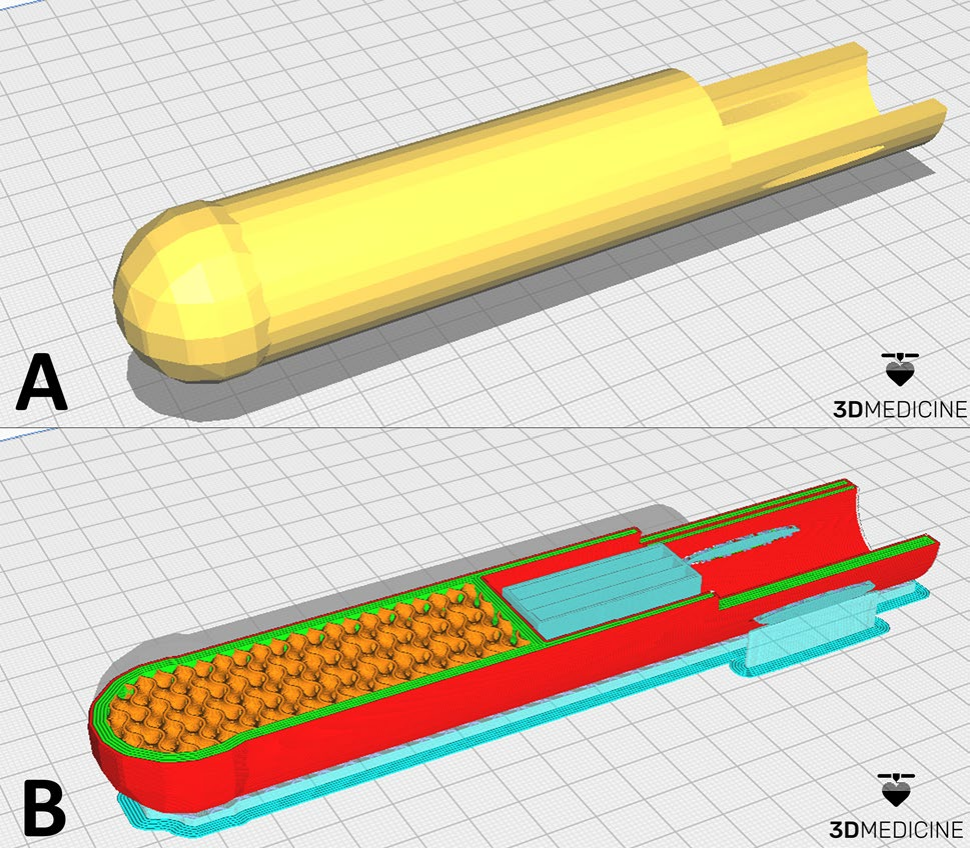
Digital 3D model of the posterior limb prosthesis of seriema (*Cariama cristata*—case report 1) measuring 32.0 × 32.2 × 171.5 mm imported into Ultimaker Cura® 5.3.0 slicer software. (**B**) Preparation and slicing of the model for 3D printing with preview of wall thickness (red and green), gyroid-shaped filling pattern (orange) and support struts (blue). (Digital model courtesy of 3D Medicine^©^—contato@3dmedicine.com.br).

The digital models of the prostheses were imported and “sliced” using the same basic settings, with specifications according to the geometry. The main parameters were as follows: printing nozzle—0.4 mm, layer height—0.12 mm, printing speed—40 mm/s, filling in gyroid format and printing temperature—205 °C (Fig. 1).

For these case reports, the material used for 3D printing was filaments made from thermoplastic *polylactic acid*, or PLA, a biodegradable and bioabsorbable polymer produced from organic sources such as corn starch, tapioca roots or sugar cane^16^. In only one case (case report 1), reinforcement structures were added to the prosthesis, in the areas of greatest impact and tension. As the aim was to add greater physical strength, these structures were printed separately using another material, p*olyethylene terephthalate glycol* (PET-G), a thermoplastic polyester, obtained from the recycling of recyclable plastic bottles, that offers greater physical and chemical resistance^23^.

Historically, PLA and ABS (acrylonitrile butadiene styrene) have been the two main polymers used worldwide, however, there is a constant search for new products and materials that offer different physicochemical characteristics of interest, such as heat and mechanical resistance, better layer adhesion (isotropy), flexibility and improved printability^15^.

Nowadays, other filaments are available in the growing market of new products for 3D printing, such as ABS (a commodity plastic, with better mechanical and thermal properties than PLA), nylon, polycarbonate (PC) and thermoplastic polyurethane (TPU) a rubber-like, flexible elastomer that can bend and compress. The choice of filament for printing is dynamic and considers the physical–chemical characteristics of the material, the purpose and usefulness of the printed piece, the type of 3D printing technology, the possible combination of materials and the geometry of the digital model^16^.

After 3D printing, the prostheses were finished by removing the support struts and smoothing out uneven surfaces using 80- to 120-grit sandpaper. A layer of protection foam was added according to the need and anatomical characteristics of the bird to avoid abrasive damage to the tissues in the amputated or malformed area.

### Ethics approval and consent to participate

The reported cases were conducted at the Veterinary Clinic of the University Center of João Pessoa (UNIPÊ) in João Pessoa, a registered educational institution (No. 02.4456.2022) at the National Council for the Control of Animal Experimentation (CONCEA), and at Zoobotanical Park Arruda Câmara "Parque da Bica" in João Pessoa, Brazil, institution registered at the Brazilian Institute of the Environment and Renewable Natural Resources (IBAMA) under number 236567.

## Results

### Case report 1—3D prosthesis for amputated hindlimb of red-legged seriema (*Cariama cristata*)

Seriemas are birds of the family Cariamidae—Bonaparte, 1853^24^ found in Argentina, Paraguay, Uruguay, and Bolivia—and are present throughout Brazil, except in the largely forested regions of the Amazon. As adults, the birds are approximately 90 cm long and weigh an average of 1.4 kg. They have wide wings and a long tail, having a gray plumage with a certain brown or yellowish hue, in addition, both the beak and the legs have a strong red color, hence the characteristic name of "red legs"^25^.

The present case report was conducted in the Zoobotanical Park Arruda Câmara "Parque da Bica" in João Pessoa, Brazil. A free-living adult female seriema (*Cariama cristata*), was rescued and taken to the park, after being found to have serious injuries (probably from being run over on the road) that led to the amputation of the left hind limb^26^.

Currently, the animal is approximately two years old, with no other alteration noteworthy, but since the amputation (one year earlier), the animal has had difficulties in locomotion, especially in the sudden and rapid movements characteristic of the species with terrestrial habits. The responsible veterinarians contacted the Brazilian company 3D Medicine^©^ (@3dmedicine.br), for the development of a 3D socket prosthesis for the seriema, through the support program for the development of new technologies for veterinary sciences.

**Figure 2:**
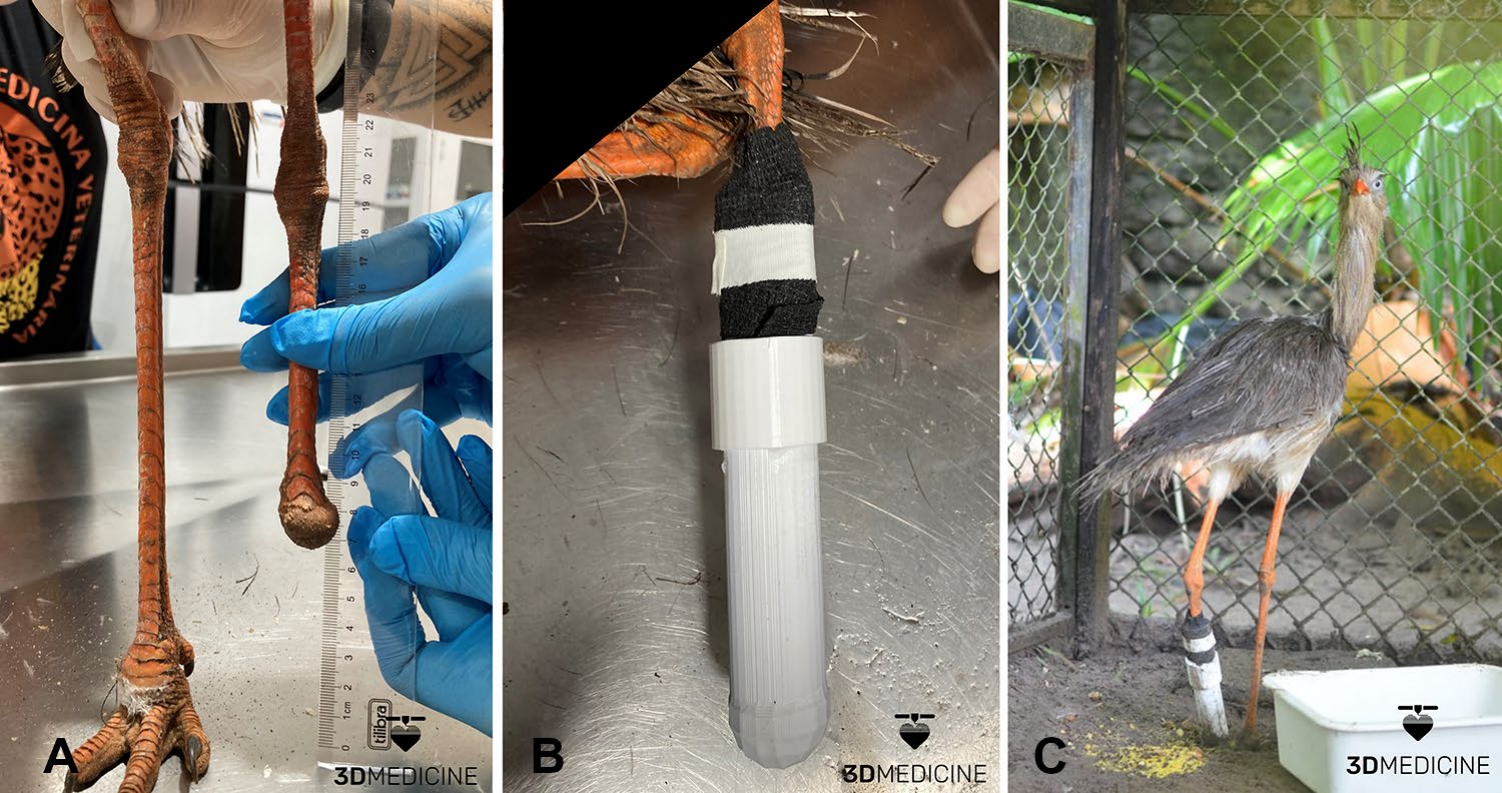
3D prosthesis for amputated hind limb of seriema (*Cariama cristata*); (**A**) Measurement of the amputated stump in comparison with the contralateral limb with alignment of the joints, height to the ground, diameter and height of the stump for fixing the 3D model; (**B**) 3D prosthesis fixed on the hind limb with a black compressive bandage and medical adhesive tape, also evident the reinforcement ring below the bandage and the sphere base before the coating with nonslip rubber; (**C**) Photographic record of the first contact with the 3D prosthesis in an adapted aviary, the bird resting both limbs on the ground, with alignment of the "knee" joints and more comfortable body weight distribution. (Photos courtesy of 3D Medicine^©^—contato@3dmedicine.com.br).

Working together with the Zoobotanical Park Arruda Câmara multidisciplinary team, a physical assessment of the animal was carried out with the measurement of all the information necessary for the construction of the digital model and 3D prosthesis (Fig. 2A). Using measurements collected from the bird, a digital prototype was built simulating the anatomical structure of the bird's foot with digits composing the base that touches the ground. However, after virtual simulations, it was observed that the presence of digits and claws, as they are not articulated, could hurt the animal, especially when perching.

Based on these considerations, the model was virtually corrected. It was possible to maintain the characteristics of the long and thin legs of seriemas, but with a spherical base at the distal end and a firm structure for fixing the amputated stump. The 3D digital model has a long, cylindrical structure, entirely filled with a gyroid pattern, five layers on the wall (total thickness of 2.0 mm) and a C-shaped structure at the proximal end for fixation with compressive bandage (Fig. 1A,B).

The digital model was materialized using the Creality CR10-Smart 3D printer (Creality 3D Technology Ltd., Shenzhen, China) with a 0.4 mm print nozzle. For this 3D prosthesis, white PET-G filament (3D Lab Industria Ltd, Belo Horizonte, Brazil) was used, a material with better mechanical and thermal resistance. In addition, a white PLA ring was placed in the proximal region to provide greater resistance in the model fixation area. In the end, the 3D prosthesis weighed 88 g.

The contact area with the stump was coated with protective material and the base was covered with nonslip rubber. The 3D prosthesis was fixed with a compressive bandage after physical restraint of the awake animal (Fig. 2B). Immediately after fixation, the bird used the prosthesis to support and balance its body weight, perform elaborate movements such as climbing on rocks and was able to eat without problems (Fig. 2C).

The animal was housed in an adapted vivarium for 7 days for observation and adaptation. After this period of getting used to the prosthesis, the bird was reintroduced into the usual aviary in the Zoobotanical Park. It is worth mentioning that until the writing of this report, the seriema has had the prosthesis for 2 months of continuous use without complications and is still under observation.

During the period of use of the prosthesis, the amputated stump was evaluated and cleaned with soap and water once a week to identify possible support injuries caused by the use of the prosthesis. The padded covering material of the prosthesis was changed every month of use and the base that touches the ground was assessed for the need for repair, however, no significant wear of the prosthesis was observed, probably due to the soft, low-abrasive earthen floor.

The process of adapting and monitoring the bird was carried out by the multidisciplinary team at Parque da Bica, which includes veterinarians, biologists and zootechnicians, with the supervision of 3D Medicine^©^ technical support.

### Case report 2—3D prosthesis for amputated hindlimb of lovebird *(Agapornis roseicollis)*

A 6-month-old *Agapornis roseicollis*, measuring 14 cm and 46 g of body weight, was admitted to the Veterinary Clinic of the University Center of João Pessoa (UNIPÊ) in João Pessoa, Brazil for clinical evaluation and routine exams. The bird had a history of strangulation injury that led to the amputation of the left limb at the level of the distal epiphysis of the tibiotarsus bone^26,27^. The owner reported that the animal had difficulty perching, walking, and standing, especially when going to feed. More accessible feeders and drinkers were adapted, but without much success.

During the general clinical examination, it was possible to observe that the amputation area was healed, but with a small new lesion in the scar tissue, probably due to the recent attempts of support on the ground. The specific evaluation of the locomotor apparatus confirmed what was reported by the tutor regarding locomotor difficulties.

A custom-modeled 3D prosthesis was recommended for the amputated stump, with the aim of protecting the area against new injuries and providing support for the body structure in a balanced way, bringing more comfort and quality of life to the animal.

The first step in the elaboration of the digital model of the prosthesis was to collect the measurements of the amputated and contralateral limb of the bird, using a digital caliper (Parkside, Stockholm, Sweden). With the animal awake and correctly restrained, measurements of the size of the amputated limb, distance to the ground, diameter at different points and comparison with the contralateral limb were performed by the responsible veterinarian.

**Figure 3:**
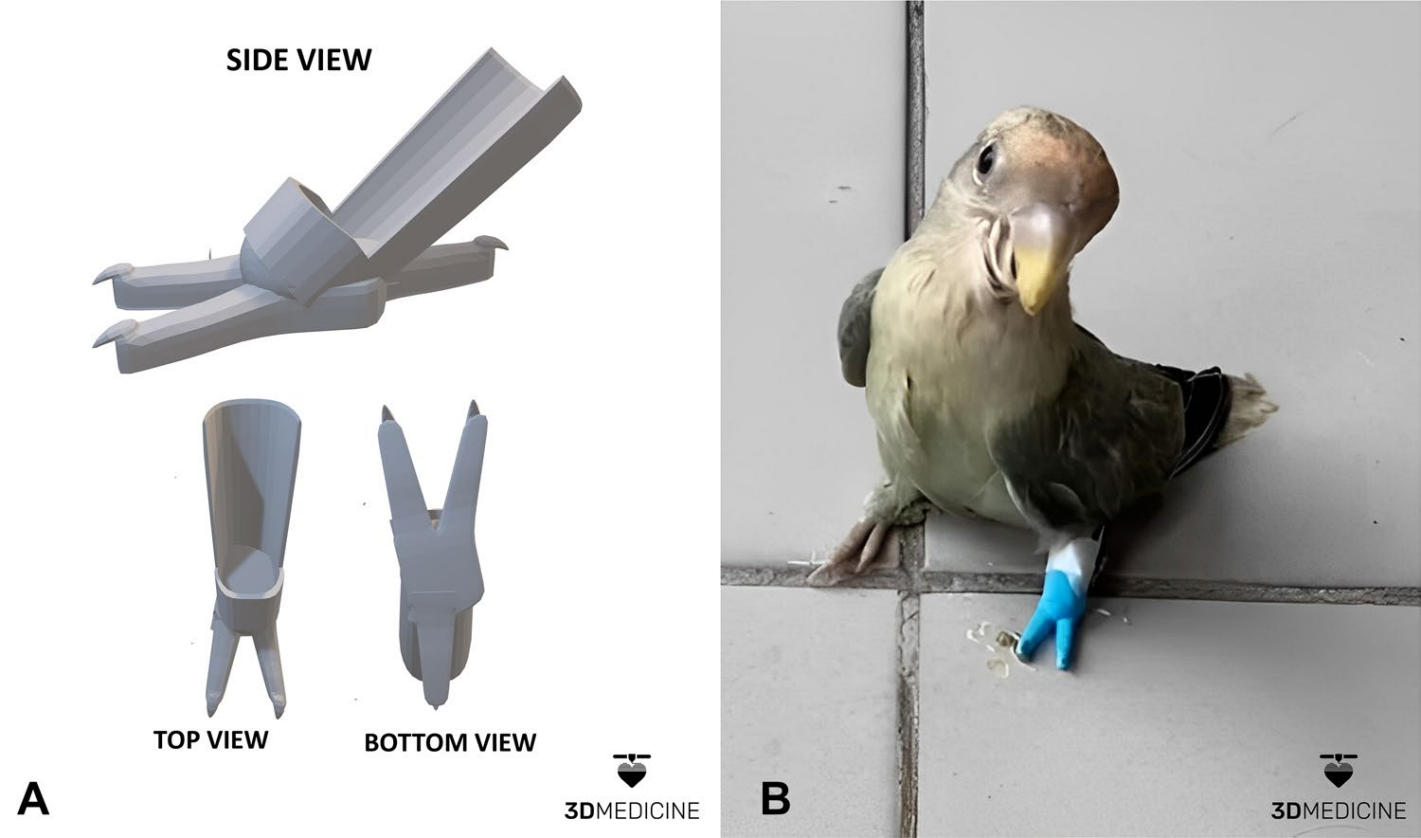
(**A**) Custom-made virtual model of a 3D prosthesis for *Agapornis roseicollis* with hind limb amputation; (**B**) Photograph captured immediately after the first fixation and interaction with the 3D prosthesis, showing the support of the amputated limb on the prosthesis, division of body weight into two bases, better stabilization of the animal and adequate reproduction of digital measurements in the 3D physical model. (Photos courtesy of 3D Medicine^©^—contato@3dmedicine.com.br).

The values in millimeters (mm) were used as parameters in the Blender® software for modeling the virtual prosthetic structure according to the needs and anatomical characteristics of the bird (Fig. 3A). The digital model was printed by the Brazilian company 3D Medicine^©^ (@3dmedicine.br), through the support program for the development of new technologies for veterinary sciences.

Using the Creality CR10-Smart 3D printer (Creality 3D Technology Ltd., Shenzhen, China) with a 0.4 mm print nozzle, the model was printed with blue PLA filament (3D Lab Industria Ltd, Belo Horizonte, Brazil). After removing the supports and finishing, the 3D prosthesis weighed less than 1 g. The model was coated internally with protective material and fixed to the *Agapornis roseicollis* with medical adhesive tape (Fig. 3B). The time of use of the prosthesis was gradually increased until complete adaptation, the process was conducted by the tutor and supervised by the veterinarian.

The bird used the prosthesis for one month until the present writing of the manuscript, even without alterations or problems, long-term evaluations of the adaptation are necessary.

### Case report 3—3D prosthesis for malformed left leg of red-rumped (*Psephotus haematonotus)*

A 4-month-old male *Psephotus haematonotus*, weighing 42 g, was admitted to the Veterinary Clinic of the University Center of João Pessoa, in João Pessoa, Brazil with difficulties in food intake and progressive weight loss. The bird was born with an almost complete malformation of the beak (agenesia), in addition to a deformity in the left leg at the level of the diaphysis of the tibiotarsus and fibula bones^26,27^.

The observed malformations result in difficulties in locomotion and proper feeding, either due to difficulty in standing up to feed or the absence of a functional beak. To facilitate the bird to obtain food, a soft and easy-to-eat food diet was indicated. In addition, more accessible feeders and drinkers were adapted in the aviary.

The physical evaluation confirms what was reported by the tutor regarding difficulties in locomotion, especially in landing and perching. Other than that, no other noteworthy symptoms were identified. Similar to the one used by lovebird (case report 2), a custom-modeled 3D socket prosthesis was recommended for the malformed red-rumped leg for this case. The purpose of this type of prosthesis would be to support the animal's body structure, divide the body weight, avoid overloading the contralateral limb and facilitate the development of simple and essential movements for the animal.

To collect the reference measurements, the animal was adequately physically restrained, and the malformed leg was measured using a digital caliper (Parkside, Stockholm, Sweden). In addition to the anatomical evaluation of the structures present in the malformed leg, values for the size of the remaining leg, height to the ground, diameter and comparison with the contralateral leg were collected.

**Figure 4:**
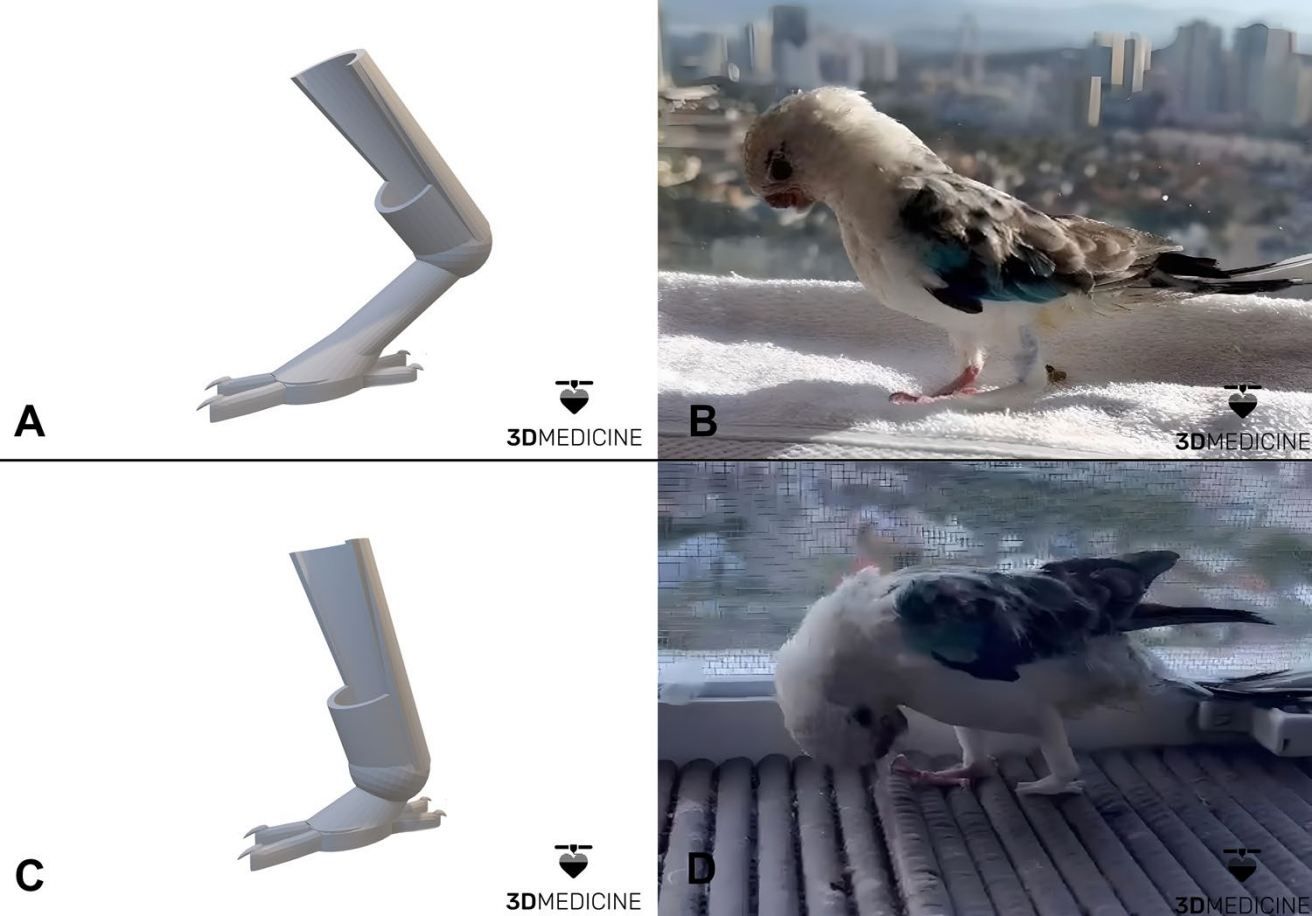
Custom-made virtual model of a 3D prosthesis for the malformed leg of Red-ramped (*Psephotus haematonotus)*. Long angled model used in the first test (**A**), photographic record immediately after the first fixation (**B**). Short, aligned model designed to correct the first test considerations (**C**), photographic record immediately after fixation of the 3D prosthesis (**D**). (Photos courtesy of 3D Medicine^©^—contato@3dmedicine.com.br).

The measurements were used as a reference for making the digital model in the 3D modeling software, Blender®. A study of the animal's behavior was added to the anatomical data to create dynamic 3D digital models that could offer a better quality of life for the bird (Fig. 4A–C).

The virtual 3D model was materialized into a physical model by 3D printing technology using a Creality CR10-Smart 3D printer (Creality 3D Technology Ltd., Shenzhen, China) and white PLA filament (3D Lab Industria Ltd, Belo Horizonte, Brazil). The 3D printing service was a collaborative action with the Brazilian company: 3D Medicine^©^(@3dmedicine.br), through the support program for the development of new technologies for veterinary sciences.

For the materialization of the 3D exo prosthetic leg prototype, the same printing settings as the previous cases were used, with some adaptations regarding the printing speed and positioning of the model on the printing table. Due to the height from the apex of the malformation to the floor, a taller prosthesis would be necessary.

Initially, a model simulating the angle of the contralateral limb was used (Fig. 4A). In the first test, due to the absence of articulated movement of the prosthesis, it was difficult for the bird to support itself and balance the weight, even very light the prosthesis was still long and rigid, especially due to the absence of joint movements of the "knee" and "ankle" (Fig. 4B). Furthermore, a long and angled prosthesis, even simulating the anatomy of the contralateral limb, increases the risk of the bird hooking the prosthesis on some structure in the aviary and becoming trapped.

On top of the questions scored in the first test, the angled model was discarded and a shorter model with a right angle was used (Fig. 4C). After printing, the prosthesis was coated internally with protective material and fixed to the animal with medical adhesive tape, making handling easier for the tutor. Immediately after fixing the second 3D model, the animal supported the leg on the prosthesis, dividing the body weight and relieving the load on the contralateral limb (Fig. 4D). When eating and landing from a short flight, it was also possible to observe the use of the prosthesis to support the malformed limb.

The adaptation process was conducted by the tutor under veterinary supervision. The time of use of the prosthesis was progressively increased during the day, however, due to the risk of the animal getting hooked in the aviary, the tutor prefers not to leave the prosthesis fixed overnight.

The bird has been using the prosthesis for three weeks at the time of writing the manuscript, the owner reported that the bird demonstrates a better distribution of body weight when resting on the prosthesis, avoiding overload in the control lateral limb. However, the details of adaptation for long-term use and the impact on the rest of the locomotor apparatus and general health are still unknown.

## Discussion

In avian medicine, regardless of the species, birds are exposed to malformations and acquired injuries (strangulation injuries, accidents, neoplasms), which lead to the amputation of a part of the body structure. Structural changes compatible with life, whether congenital or acquired, are most often observed in the hind limbs, wings, and beaks^6,8,9^.

Custom-designed prostheses are an alternative for birds with deformities and amputations, however some characteristics for these animals must be considered in comparison to the development of prostheses in mammals. Specifically for hind limb prostheses, most birds are classified as digitigrade, an animal that supports the toes (phalanges) on the ground, the leg has fused bones (tibiotarsus, tarsometatarsus) and is commonly thin and long with little muscle tissue, which directly reflects on the fixation method, resistance, and biomechanics of the prosthetic structure. The bird's behavioral habits, as well as the habitat where the animal will be located, must also be considered^14,26^.

Three cases of structural alterations related to the locomotor apparatus of domestic and wild birds was reported in this study. The animals had locomotor problems and were indicated to use orthopedic exo prostheses in order to improve their quality of life. Two cases were caused by physical trauma followed by amputation of the hind limb (cases 1–2) and one case was caused by congenital malformation (case 3).

For avian medicine, two main methods can be used for prosthesis production and fixation: (1) conventional socket prostheses where the limb is fitted into the cup-shaped structure of the prosthesis with external fixation^11,12^; and (2) models fixed by osseointegration, a technique that uses an intramedullary pin incorporated into the bone of the amputated extremity to fix the external prosthesis, a technique described by Hochgeschurz and collaborators^13^, for the prosthesis of a one-year-old female bearded vulture (*Gypaetus barbatus*) with an amputation at the tarsal-metatarsal level due to a strangulation injury.

In this study, the conventional socket prostheses method was used in the three cases. The models were produced by computational design and printed by fused deposition modeling (FDM) an additive manufacturing technique. The combination of virtual model development methods and 3D printing of the prototype, allows the manufacture of light, resistant and tailored models, using materials with different physical–mechanical characteristics^18^.

These tools have great potential for application in different areas of veterinary medicine, in addition to being under the scope of precision medicine, creating personalized models for animal patients with significant anatomical differences^28,29^.

For birds, the socket prostheses are easier to apply compared to the osseointegration method, as there is no need for an invasive surgical procedure and recovery/healing period, making the process cheaper and more accessible. These prostheses are easily removable, facilitating handling for bird adaptation, cleaning, and routine care.

However, in addition to the lack of joint movement, another major limitation of socket prostheses for birds is the fixation method. For this type, the amputated stump (often thin and small) must fit into the "cup" structure of the socket prosthesis, secured by adhesive tape or bandage. In this way, there is the possibility of the prosthesis falling off, or dislocating and loosening.

Galicia and collaborators describe a case of 3D printing of a socket prosthesis for the leg of a Red-lored Amazon Parrot (*Amazona autumnalis*), using the same printing and fixation methods in this study. The authors describe that in a period of four months, three different 3D prosthesis models were used, with corrections in the structure and fixation methods according to the tests with the bird. A similar process was observed in case 3 of this study, where a model was tested and due to the animal's characteristics, another 3D prosthesis had to be made to correct the components detected in the first evaluation^12^.

Socket prostheses have also been described in larger and heavier land birds, as reported by Ranganathan and collaborators, for modeling and printing a 3D prosthesis for a peacock, the bird using the prosthetic leg was able to stand still and perform dynamic movements, enabling a significant increase in quality of life^11^.

The present case reports series describes the use of 3D prostheses, with socket model fixation for three different species of birds in Brazil. To the author's knowledge, no report of the use of this technology to develop prostheses for the described species has been published in refereed journals. In addition, this is the first description of the application of 3D prostheses in very small birds weighing less than 50 g and prostheses weighing approximately one gram, which reinforces the accuracy of 3D printing and the possibility of application in passerines and smaller birds.

## Conclusion

This study describes the use of three-dimensional technology for modeling and printing prostheses for domestic and wild birds with locomotor disorders due to amputations or malformation of the hindlimbs in Brazil. The 3D prostheses developed were accessible, light, resistant and significantly increased the birds' quality of life in a noninvasive way. 3D technology is in constant development, with valuable tools for use in avian medicine and other areas of veterinary medicine, such as in the manufacture of orthopedic and structural prostheses, virtual or physical surgical planning, production of models for anatomical studies, surgical tools, and other applications to be explored. However, few veterinary professionals explore 3D technology or are aware of the application possibilities, therefore, more dissemination and refinement of methods are needed.

## Data Availability

3D models and the datasets used during the current study are available from the corresponding author upon reasonable request.

## Abbreviations

3D: Three-dimensional
FDM: Fused deposition modeling
CT: Computerized tomography
MRI: Magnetic resonance imaging
Fps: Frames per second
mm: Millimeter
CNC: Computer numerical control
STL: Stereolithography
AM: Additive manufacturing
mm/s: Millimeters per second
PLA: Polylactic acid
PET-G: Polyethylene terephthalate glycol
TPU: Thermoplastic polyurethane
Kg: Kilogram
Cm: Centimeter
